# Orthogonality catastrophe and quantum speed limit for spin chain at finite temperature

**DOI:** 10.1038/s41598-022-09010-5

**Published:** 2022-03-23

**Authors:** Zheng-Rong Zhu, Qing Wang, Jian Zou, Bin Shao, Lian-Ao Wu

**Affiliations:** 1 School of Physics, Beijing Institute of Technology, Beijing, 100081 China; 2 Yangtze Delta Region Academy of Beijing Institute of Technology, Jiaxing, 314019 China; 3 Ikerbasque, Basque Foundation for Science, 48011 Bilbao, Spain; 4 Department of Physics, The Basque Country University (EHU/UPV), PO Box 644, 48080 Bilbao, Spain

**Keywords:** Quantum information, Theoretical physics

## Abstract

We present an interesting relationship between the orthogonality catastrophe (OC) and the quantum speed limit (QSL) for a spin chain with uniform nearest neighbour couplings perturbed by an impurity spin. We thoroughly study the catastrophic QSL that specifies a bound on the evolution time between the initial and final states and in this respect, link it to the emerging OC effect. It is found that the speed of state evolution subtle but fundamental, and the bound characterized by QSL shows the same behaviours as the OC effect in the thermodynamic limit. It allows us to reveal some universal properties, in particular finite temperature effects. Significantly, the threshold of temperature and system size is clearly demonstrated for the QSL under finite temperature.

## Introduction

Contemporary research on the orthogonality catastrophe (OC)^1^ dates back to the original definition proposed by Anderson and this concept has boosted extensively interests over the recent years. This is attributed to the experimental development in ultracold atoms where desired many-body states^2,3^ and amiable manipulated Hamiltonians are created. These are crucial for motivation on studying quantum dynamics and control. Furthermore, the technique advance of both Ramsey interference^4^ and radio-frequency spectroscopy paves the way for exploring OC in the many-body fermionic systems quenched by impurities from the time and frequency domain respectively.

The essence of OC lies in the overlap between the initial unperturbed state and the final state perturbed by the local impurity, which vanishes with the system size *N*. Such an effect in many-body systems has been broadly probed in different scenarios, by evaluating the dynamics of correlation functions^5^, exploring statistics of work^6^ introduced into the system in quantum spin models^7^, analyzing the energy distributions in x-ray edge problems^8^, relating it to decoherence of impurity and non-Markovian dynamics^9^ and understanding quantum adiabaticity breakdown^10^.

Interestingly, the overlap plays an important role in the promising concept of the quantum speed limit (QSL) which originates from the Heisenberg uncertainty principle^11^ whose central task is to find a bound on the minimum evolution time between two quantum distinguishable states. Also, as a consequence of its simplicity in describing the quantum many-body systems, the QSL has been applied to many aspects in quantum techniques, such as quantum computation^12^, quantum state transfer^13^, as well as optimal control^14^ of quantum open systems. Therefore, it would be of great interest to explore the relation between the QSL and the OC. Recently, a connection between the dynamics of OC and QSL has been established in Ref.^15^, where the QSL is shown to be capable of bounding the time to reach OC as long as the variance of the quenched Hamiltonian under the initial ground state scales with the system size, for instance in trapped fermi gas and interacting LMG model. However, the correlation between the OC and the QSL is hardly considered in the prototypical scheme of the nearest-interacting spin chain, a typical testing bed for various physical phenomena, such as quantum phase transition at critical points, coherence loss in quantum systems and so on. In this paper, we study this typical spin system with a single qubit impurity switched on initially. We showcase the OC effect and link it to the maximum rate of quantum many-body evolution signalled by the QSL time. Then, specifying a universal relation in particular an extension to finite temperature effect, where we demonstrate the threshold of temperature and system size *N*.

The rest paper is organized as follows. Firstly, we present the relation between the OC and the QSL and generalize the pure state results to the finite temperature case by virtue of a bound on Bures angle based on density operators. Secondly, we briefly review the spin chain model of interest and obtain the exact expressions of the fidelity for both cases of initial ground and thermal states. Thirdly, we show numerical comparison and analysis between the OC and the QSL. Here we emphasize the finite temperature effect. At last, conclusions are drawn.

## Orthogonality catastrophe and quantum speed limit

The OC, in Anderson’s original work^15^, was involved with stationary states, while the quenched many-body state, in reality is time-dependent. In this aspect, the dynamical orthogonality after the perturbation switched on has to be considered and characterized by a dynamical overlap, read as

1 $$\begin{aligned} \chi \left( t \right) = \left\langle \Psi \right| {e^{i{H_{f}}t}}{e^{ - i{H_{i}}t}}\left| \Psi \right\rangle \end{aligned}$$

with ${H_f}$ and ${H_i}$ representing the final quenched and initial Hamiltonian, respectively. Assume $\Psi$ as the eigenstate of ${H_i}$, eigenvalue being $E_i$, ${\chi \left( t \right) }$ is thus reduced to $\chi \left( t \right) = \left\langle \Psi \right| {e^{i{H_f}t}}\left| \Psi \right\rangle {e^{ - iE_it}}$. Since its association with the fidelity $F\left( t \right) = {\left| {\chi \left( t \right) } \right| ^2}$ which occupies the centre of the QSL and furthermore, is also the so-called Loschmidt echo^16,17^ in the fidelity program, we will adopt the notation of fidelity instead in rest of the paper.

The QSL captures the minimum time that quantum system requires for evolving between two states. We interpolate the Bures angle^18^ in order to establish a connection with OC, since they both are related to the overlap function (1).

2 $$\begin{aligned} {\mathscr {L}}\left( t \right) \equiv \arccos \left| {\chi \left( t \right) } \right| = \arccos \left| {\left\langle {{{\psi _t}}} |{{{\psi _0}}} \right\rangle } \right| , \end{aligned}$$

where $\left| {{\psi _t}} \right\rangle = {e^{-i{H_f}t}}\left| \Psi \right\rangle$ and $\left| {{\psi _0}} \right\rangle = \left| \Psi \right\rangle$. Combining with a lower bound in Eq. (2) based on quantum fisher information for an estimation of time^18^, one has

3 $$\begin{aligned} {\mathscr {L}}\left( \tau \right) \le \frac{1}{2}\int _{0}^{\tau } {dt\sqrt{ {\mathscr {I}}} }, \end{aligned}$$

where the quantum fisher information ${\mathscr {I}}$ can be exactly computed in pure states and unitary evolution as ${{\mathscr {I}}} = 4\left( {\left\langle {H_f^2} \right\rangle - {{\left\langle {{H_f}} \right\rangle }^2}} \right) = 4\Delta H_f^2$, with average over the initial state, the fidelity is hence fully restrained by the time average of the variance of ${H_f}$ which gives rise to a connection between QSL and OC. In addition, a bound on the fidelity was derived with respect to time-dependent quenched Hamiltonian^19^. Since ${H_f}$ in our system is time independent, we then easily come to the significant formulation linking QSL and fidelity,

4 $$\begin{aligned} \tau \ge {\tau _{QSL}} = \frac{{\arccos \left| {\chi \left( \tau \right) } \right| }}{{\Delta {H_f}}}. \end{aligned}$$

Here, ${\Delta {H_f}}$ viewed as the maximal evolution rate of system $v_{QSL}$ is a function of the system size *N*, when ${\Delta {H_f}}$ increases monotonically with *N*, then the minimum time for the system state to evolve into an orthogonal final state consequently vanishes. On the contrary, if ${\Delta {H_f}}$ decays with the size *N*, ${\tau _{QSL}}$ increases with *N* accordingly, and the time to hit the orthogonality becomes infinite. On the other aspect, this confirms the decisive relation between QSL and OC. Additionally, Eq. (1) is closely related to the work done in quenching the system^20,21^, such that the QSL time also implies thermodynamic significance in physics.

We now extend the above ideas to the case with an initial thermal state, which allows one to analyse the thermal effects. However, there unfortunately exists a plight where inequality (3) cannot be analytically given, since the quantum fisher information in the initial mixed-state is hard to explicitly calculate and a purification procedure for large number of particles is required. The way to circumvent this problem (e.g., see Ref.^22^) is to employ a bound for thermal states in terms of Bures angle based on the density operator, which can be cast as

5 $$\begin{aligned} {\mathscr {L}} \left( \rho _1, \rho _2 \right) \le \arcsin \left( \sqrt{\beta t} \root 4 \of {-2\langle [H_i,H_f]^2}\rangle _{\beta }\right) , \end{aligned}$$

where the Bures angle for the mixed state is defined as ${\mathscr {L}}\left( \rho _1, \rho _2 \right) \equiv \arccos \ tr \sqrt{\sqrt{\rho _1}\rho _2 \sqrt{\rho _1}}$, $\rho _1,\rho _2$ correspond to the initial and final evolved density operators. $F(t)=tr \sqrt{\sqrt{\rho _1}\rho _2 \sqrt{\rho _1}}$ is considered as the mixed-state fidelity of the spin chain system. To make Eq. (5) explicit for the further calculation, it can be converted, after a straightforward transformation and in a similar form as Eq. (4) to

6 $$\begin{aligned} \tau \ge {\tau _{QSL}} =\frac{\sin ^2(\arccos \ tr \sqrt{\sqrt{\rho _1}\rho _2 \sqrt{\rho _1}})}{\beta \sqrt{-2\langle [H_i,H_f]^2 \rangle _{\beta }}}, \end{aligned}$$

where the temperature dependent denominator in Eq. (6) can be seen as the evolving speed $v_{TQSL}$, and the exact formulation in our model will be given in a later section. With all the above results, it is of great convenience for us to tackle problems of a specific model like spin chain system and demonstrate the relation between OC and QSL for different choices of initial states. We emphasize here that the temperature effects show considerable characteristics.

## The specific spin chain model and quench dynamics

We now focus on a distinct system described by an one-dimensional nearest neighbour interacting spin chain, which is suddenly quenched by single spin^23,24^ taken as an impurity. In the standard model, the total postquench Hamiltonian of the system is given by ${H_f} = {H_i} + {H_I}$, where ${H_i}$ refers to the initial Hamiltonian before switching on the perturbation, while ${H_I}$ is the interaction between the surrounding spin chain and impurity which is turned on at *t* = 0, with

7 $$\begin{aligned} \begin{aligned} {H_i}&= - J\sum \limits _{l=1}^N {\left( {\frac{{1 + \gamma }}{2}\sigma _l^x\sigma _{l + 1}^x +\frac{{1 - \gamma }}{2}\sigma _l^y\sigma _{l + 1}^y + \lambda \sigma _l^z} \right) }, \\ H_I&=J\delta \sigma ^z \sum \limits _{l=1}^N \sigma _l^z, \end{aligned} \end{aligned}$$

where the parameters ${\lambda }$ and ${\gamma }$ denote intensities of the external magnetic field and the anisotropy values to distinguish various types of spin models, respectively. Here, $\gamma =0$ corresponds to an XX model, $0 \le \gamma \le 1$ characterizes the degree of anisotropy, and the critical magnetic field $\lambda _c=1$ is unchanged regardless of the value of $\gamma$. The operators ${{\sigma ^z}}$ and ${\sigma _l^{n}}$, $n = \left\{ {x,y,z} \right\}$ are Pauli matrices of the single spin and its environment. $\delta$ is a coupling constant (typically weak) and the exchange energy *J* is set to be one for simplicity. Suppose that the impurity spin is in a superposition state $\left| \varphi \right\rangle = {c_g}\left| g \right\rangle + {c_e}\left| e \right\rangle$, $\left| g \right\rangle = \left( 0 \right. ,{\left. 1 \right) ^T}$ and $\left| e \right\rangle = \left( 1 \right. ,{\left. 0 \right) ^T}$, where coefficients ${c_g}$ and ${c_e}$ are normalized. In terms of the impurity spin state, the postquench Hamiltonian is rewritten as

8 $$\begin{aligned} {H_f} = \left| g \right\rangle \left\langle g \right| \otimes H_f^g + \left| e \right\rangle \left\langle e \right| \otimes H_f^e, \end{aligned}$$

wherein

9 $$\begin{aligned} \begin{aligned} H_f^g&= \left\langle g \left| {H_f} \right| g \right\rangle = {H_i} - J\delta \sum _l^N {\sigma _l^z}, \\ H_f^e&= \left\langle e \left| {H_f} \right| e \right\rangle = {H_i} + J\delta \sum _l^N {\sigma _l^z}. \end{aligned} \end{aligned}$$

Here ${H_f^{\alpha }}$ can be diagonalized in a standard procedure by using Jordan–Wigner transformation which maps the $\frac{1}{2}$ spins into spinless fermions, Fourier transformation converting the Hamiltonians into k-space form, and Bogoliubov transformation under the imposed period boundary condition to the simplified forms^25^

10 $$\begin{aligned} \begin{aligned} H_f^\alpha&= \sum _{k=1}^M {H_f^{k,\alpha }} \\&= \sum _{k=1}^M {2\Omega _k^\alpha \left( {b_{k,\alpha }^\dag b_{k,\alpha }^{} - \frac{1}{2}} \right) }, \end{aligned} \end{aligned}$$

where $\alpha = g,e$. The quasiparticle energy spectra ${\Omega _k^\alpha }$ in Eq. (10) are given by

11 $$\begin{aligned} \Omega _k^\alpha =J \sqrt{\gamma ^2 \sin ^2 \frac{2\pi k}{N}+(\cos \frac{2\pi k}{N}-\lambda _{\alpha })^2}, \end{aligned}$$

with $\lambda _g = \lambda +1, \lambda _e =\lambda - 1$, and $b_{k,\alpha }=\cos \frac{\theta _k^{\alpha }}{2} d_k - i\sin \frac{\theta _k^{\alpha }}{2} d_{-k}^{\dag }$ being the Bogoliubov transformations with the angles $\theta _k^{\alpha }=\arccos [J(\cos \frac{2\pi k}{N}-\lambda _{\alpha }) / {\Omega _{k}^{\alpha }}]$. Assume that the initial state of the surrounding spins is in ground state $\left| G \right\rangle _{\lambda }$ of ${H_i}$, the relation between the ground state $\left| G \right\rangle _{{\alpha }}$ of ${H_f^{\alpha }}$ and $\left| G \right\rangle _{\lambda }$ are directly associated by the Bogoliubov transformation,

12 $$\begin{aligned} \left| G \right\rangle _{\lambda } = \prod _{k=1}^{M} (\cos \Theta _k^{ {\alpha }}+ i \sin \Theta _k^{ {\alpha } } b_{k,{\alpha }}^{ \dag } b_{-k,{\alpha }}^{\dag })| G\rangle _{{ \alpha } }, \end{aligned}$$

where $\Theta _k^{ {\alpha }}=(\theta _k^{ {\alpha }}-\theta _k^{ \lambda })/2$, and $\theta _k^{\lambda }=\arccos [J(\cos \frac{2\pi k}{N}-\lambda ) /{\Omega _{k}^{\lambda }}]$. With all above ingredients, we aim at deriving an explicit expression for the fidelity. And we also initialize the impurity spin in ground state $\left| g \right\rangle$ for simplicity. Without loss of generality, we finally obtain the fidelity,

13 $$\begin{aligned} \begin{aligned} F\left( t \right)&= {\left| {\prod \limits _k ^{M}{{{\sin }^2}\Theta _k^{{g}} \times {e^{\left[ {-2i\left( {\Omega _k^{{g}} + \Omega _k^\lambda } \right) } \right] }} + {{\cos }^2}\Theta _k^{{g}} \times {e^{\left[ {2i\left( {\Omega _k^g - \Omega _k^{{\lambda }}} \right) } \right] }}} } \right| ^2}\\&= \prod \limits _{k = 1}^M {\left[ {1 - {{\sin }^2}\left( {2\Theta _k^{{g}}} \right) {{\sin }^2}\left( {2{\Omega _{k}^{g}}} \right) } \right] }, \end{aligned} \end{aligned}$$

where $\Omega _k^{\lambda }=J\sqrt{\gamma ^2 \sin ^2 \frac{2\pi k}{N}+(\cos \frac{2\pi k}{N}-\lambda )^2}$ refers to the excitation spectra of the undisturbed system Hamiltonian.

Next we turn our attention to calculate the exact form of the fidelity under an initial thermal equilibrium states, for the finite temperature effect is of fundamental significance in realistic physics systems. Density matrices of initial thermal state can be analytically derived as

14 $$\begin{aligned} \rho =\frac{1}{Z} \exp \left( -\beta H_i\right) , \end{aligned}$$

here $\beta ={1}/{k_B T}$ and *Z* is the partition function, where $k_B$ is the Boltzmann’s constant set as one for convenience, *T* is the temperature specified as $k_B T/J$ in the later discussions and J denotes the exchange energy used as the energy unit. Note that the partition function *Z* is determined by unperturbed Hamiltonian $H_i$,

15 $$\begin{aligned} Z=Tr\left[ \exp \left( -\beta H_i\right) \right] . \end{aligned}$$

It is natural for us to extend the notation of the survival probability to a thermal state by using the mixed-state fidelity^26^ and we can directly calculate the mixed-state fidelity as,

16 $$\begin{aligned} F( t )= & {} \prod \limits _k \left( [ 1+\cosh (2 \beta \Omega _k^{\lambda }) ]^{-1}\right. \nonumber \\&\times \left( 1+\frac{1}{\sqrt{2}}\{[1 - {{\sin }^2}( {2\Theta _k^{{g}}} ){{\sin }^2}( {2{\Omega _{k}^{g}}} )] \right. \nonumber \\&\left. \left. \times \cosh (4 \beta \Omega _k^{\lambda })+1 +{{\sin }^2}( {2\Theta _k^{{g}}} ){{\sin }^2}( {2{\Omega _{k}^{g}}} ) \}^\frac{1}{2}\right) \right) ^2. \end{aligned}$$

It is clear that in the zero-temperature limit, $\beta \rightarrow \infty$, one easily recovers the result obtained in the ground state case. By changing $\beta$, we can explore the fidelity of the initial thermal state and what role it plays in the connection between OC and QSL.

## Comparison and analysis of orthogonality catastrophe and quantum speed limit

Now we show the numerical results and analyze the relation of OC and QSL. To proceed, let us start with examining the initial ground state case. Based on the analytical discussions, an important quantity to link both of them is the energy variance of perturbed Hamiltonian and in our model, with the system being the initialized ground state of unperturbed Hamiltonian. $\Delta H_{f}$ after exactly calculations is therefore formulated as

17 $$ \Delta H_{f} = \sqrt{\sum \limits _{k = 1}^{M} {16( {\Omega _{k}^{g}}{{\cos }}\Theta _k^g{{\sin }}\Theta _k^g )^2}}. $$

**Figure 1:**
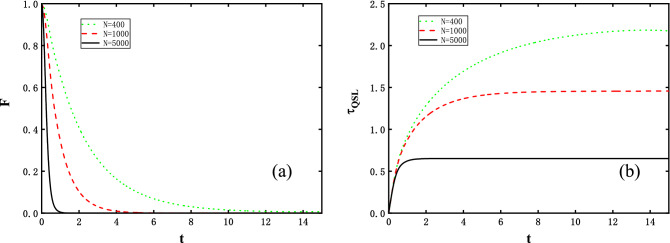
Time in units of 1/*J*, evolution of (**a**) the fidelity *F* and (**b**) the QSL time $\tau _{QSL}$, with system size *N* = 400 (green dotted line), 1000 (red dashed line), and 5000 (black solid line). The external magnetic intensity, anisotropy value, and coupling constant set as $\lambda =1$, $\gamma =1$, and $\delta =0.05$, respectively.

$\Delta H_{f}$ coincides with the analytical results derived in Ref.^27^, and for large spin system Eq. (17) scales linearly with *N*. The variance shows two different regimes characterized by the external magnetic field $\lambda$, i.e., independent of $\lambda$ for $\lambda \le 1$ and scaling as ${1}/{\lambda ^2}$ for $\lambda > 1$. Figure 1 shows time *t* functional of the fidelity and the QSL for the parameters exhibited in the caption. We observe immediately a drop in the fidelity, furthermore the larger *N* grows, the steeper curves *F* get. As such, these behaviours indicate an orthogonality to the initialized spin chain ground state after the system experiences an evolution process with the disturbance suddenly switched on. In particular, the orthogonality time gets smaller and is approaching zero with *N* increased. Such a phenomena can be verified by Fig. 1b. Correspondingly, the QSL time in Fig. 1b eventually reaches a maximal stationary value after an interval of evolution time. Meanwhile, the QSL time, in its maximum value induced by the orthogonality of two evolved states, decreases with the increased *N*. The specific feature reveals a similar OC effect that the QSL time holds and will be explicitly observed in the later analysis.

**Figure 2:**
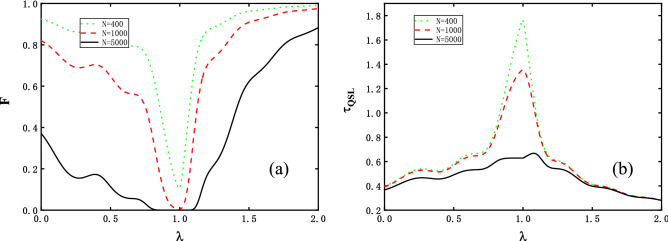
(**a**) The fidelity *F* and (**b**) the QSL time $\tau _{QSL}$ as functions of the external field $\lambda$. Each different color line corresponds to the same colored line routine as in Fig. 1, i.e. *N* = 400, 1000, 5000 for green dotted, red dashed and black solid lines respectively. Here we set a fixed actual evolution time *t* = 5, weak perturbation $\delta =0.05$, and anisotropy value $\gamma =1$.

The dependence on external magnetic field is also of interest. Figure 2 displays the variations of *F* and $\tau _{QSL}$ for the external magnetic intensity at fixed moments in time. For Fig. 2a, the decay of the fidelity is enhanced with *N* which clearly shows the OC effect at the vicinity of critical point. Moreover, the wider range of valley makes the criticality at $\lambda =1$ blurry. On the other hand, it deserves note that apart from the critical point, the two regions $\lambda < 1$ and $\lambda > 1$ exhibit a subtle phenomenon where the weaker the external field is, the more sensitive to *N* *F* is. As a result, the QSL time in Fig. 2b shows a cusp, but gets weaken due to a broader valley. For the two external magnetic field regimes, the decay of $\tau _{QSL}$ is not susceptible to *N*, since the spin chain internal nearest interaction or external strong magnetic strength are dominate^28^, and the evolving speed $\Delta H_{f}$ showing distinct behaviours in the two regimes also contributes to this phenomenon. To conclude, the emerging OC and its relation to QSL time are more apparently seen at the vicinity of critical magnetic field in comparison with far from it.

**Figure 3:**
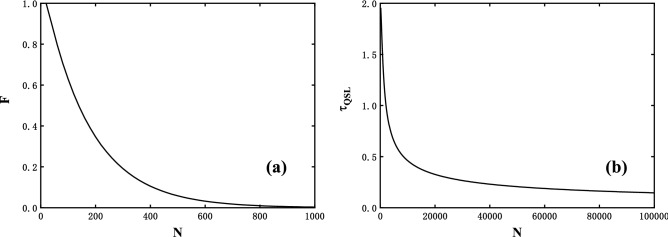
Selected fixed time instants at *t* = 5 during the dynamical evolution of the quenched spin chain at anisotropy value $\gamma =1$ and critical external field $\lambda =1$ for illustrating (**a**) the fidelity *F* and (**b**) the QSL time $\tau _{QSL}$ as functions of *N*.

Next we focus on the behaviours of both *F* and $\tau _{QSL}$ at the critical $\lambda =1$ with *N* grows, analyzed in Fig. 3a and b respectively. We find *F* decays to zero as *N* grows which is a witness of the OC, and we also see a similar behaviour $\tau _{QSL}$ holds. It allows us to reveal a universal relation and consider the Fig. 1, we conclude that an vanishing $\tau _{QSL}$ is in correspondence to an vanishing time to reach OC, i.e., the OC is a consequence of the QSL as demonstrated in Ref.^15^. Additionally, it would nevertheless note that the evolved states reach orthogonality at small *N*, the QSL time will ultimately approach to zero in the thermodynamic limits. To explain this phenomenon, we may pay attentions to the critical magnetic field where the decay of the fidelity is extremely sensitive to *N*. Meanwhile, it is also interesting to notice that the energy variance of the perturbation Hamiltonian or the evolving speed increasing slowly with *N*.

### Finite temperature effect

**Figure 4:**
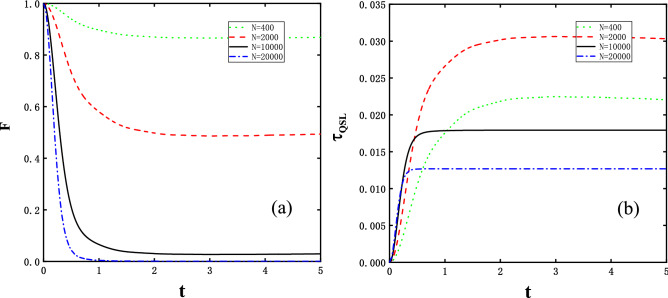
Finite temperature *T* = 0.5. (**a**) the fidelity *F* and (**b**) the QSL time $\tau _{QSL}$ as functions of time *t* at different *N* (see legend), where in both panels the coupling strength, anisotropy value and external magnetic field are constant $\delta =0.05$, $\gamma =1$, and criticality $\lambda _c=1$, respectively. It can be obviously seen that either panels show a sharp decay or increase to a stable minimum or maximum value.

The temperature effect would be of interest for a realistic system always being non-zero temperature. Similarly, the quantity $\langle [H_i,H_f]^2\rangle _{\beta }$ has to be exactly calculated to evaluate the QSL time under finite temperature, as shown in Eq. (6) and reads as,

18 $$\begin{aligned} \langle [H_i,H_f]^2 \rangle _{\beta }=\prod _{k=1} (1+\cosh (2\beta \Omega _{k}^{\lambda }))^{-1} 64\gamma ^2 g^2 \sin ^2 \frac{2\pi k}{N} \cosh (2\beta \Omega _{k}^{\lambda }), \end{aligned}$$

where the average is taken over initial thermal state. We define a quantity relating to evolving speed $v_{TQSL}=\beta \sqrt{-2\langle [H_i,H_f]^2\rangle _{\beta }}$ under finite temperatures following the routine in the case for the initial ground state. Note that the defined evolving speed scales extensively with *N*, but depends on temperature, differing from the ground state speed. Figure 4a explores the time dependence of the fidelity *F* for the size *N* in the range of 400 and 20,000. The figure shows two remarkable features: First, *F* do not decay to zero until *N* reaches a comparatively large value and similar to ground state, the fidelity *F* decays faster with *N* grows. Second, *F* decays to a minimum stationary value immediately. In contrast with the zero-temperature ground state case, the temperature factor leads to a drop in the decay amplitude of the fidelity, implying that the system requires more spins for the fidelity to be completely vanished. Furthermore, we also examine the QSL time depicted in Fig. 4b, where considerable characteristics are shown. For long driving time, the plateau in $\tau _{QSL}$ corresponds to the bottom platform in *F* approximately. The maximum stationary $\tau _{QSL}$ first increases before *N* reaches a certain value, after the threshold, $\tau _{QSL}\rightarrow 0$ as $N \rightarrow \infty$. While as to comparatively short evolving time, e.g. $t\le 1$, $\tau _{QSL}$ is somehow complicated and seems to increase with *N*.

**Figure 5:**
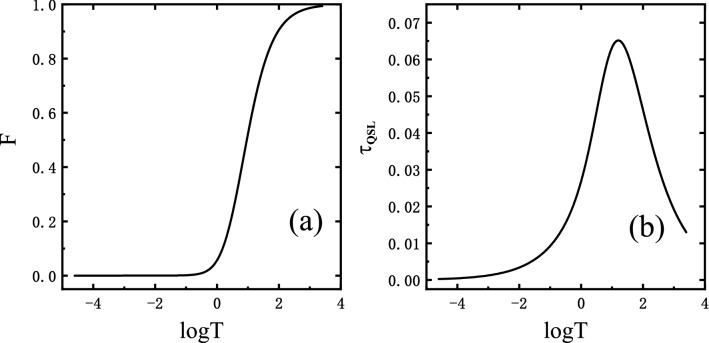
(**a**) The fidelity *F* and (**b**) the QSL time $\tau _{QSL}$ as functions of temperature at a fixed instant *t* = 5 for a environment size of $N=2\times 10^4$, with other parameters in both (**a**) and (**b**) are chosen as $\lambda =1$, $\gamma =1$, and $\delta =0.05$. Note that there are clearly anomalous phenomena that $\tau _{QSL}$ shows a cusp-like shape.

Here, we emphasize the temperature effects on OC and QSL. Figure 5 shows how the fidelity and the QSL time change with temperature for parameters shown in the caption. The decay of *F* in Fig. 5a becomes less evident with temperature, and it shows no decay for even higher temperature due to the thermal excitation in spin chain, leading to the system state approaching maximally mixed and trivial dynamics. In what follows, $\tau _{QSL}$ differs from the behaviour of *F* in that, first, as shown in Fig. 5a when the temperature is below *T* = 1 or so, it shows a plateau where the fidelity completely vanishes or the evolved states reach orthogonality, which corresponds to the increased QSL time. An increase in $\tau _{QSL}$ implies a slowdown in the orthogonal speed. Second, the QSL time $\tau _{QSL}$ shows a oscillation phenomenon with *T* grows contrary to the conventional monotonic fidelity. These are somehow counter-intuitive against the previous knowledge that system quantumness encoded in the overlap should be washed out due to the thermal excitation as temperature rises. To understand the interesting phenomenon, now we study the evolving speed $v_{TQSL}$ that is inversely proportional to temperature, i.e., the rate of system evolution is suppressed by *T*. On the other hand, the decay of fidelity in suppression under higher temperature also contributes to the aforementioned phenomenon. In brief, the temperature effects cause suppression of both evolving speed and the decay of fidelity and an explicit threshold of temperature is seen in the QSL time.

**Figure 6:**
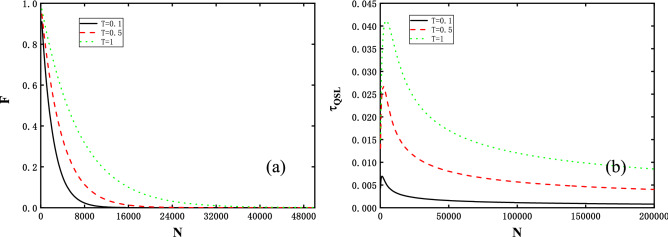
The environmental size *N* dependence of (**a**) the fidelity *F* and (**b**) the QSL time $\tau _{QSL}$ at critical point $\lambda =1$ and with the same total evolution time, anisotropy parameter and coupling constant as Fig. 5 for different temperature, i.e., *T* = 0.1, 0.5, 1 correspond to black solid, red dashed and green dotted lines respectively.

In Fig. 6, we plot the $\tau _{QSL}$ and *F* vs *N* to specify a substantial relation between the QSL time and the OC for finite temperatures. As shown in Fig. 6a, *F* at a given moment decays to zero when *N* reaches certain value as a OC witness. While the lower temperature *T* system is at, the smaller *N* the fidelity *F* vanishes at. Note that Fig. 6b also shows several interesting features: $\tau _{QSL}$ spikes at a certain critical *N* which is greatly influenced by temperature, yielding a scaling extensively with *T* behaviour. Interestingly, this phenomenon also explains why the QSL time in Fig. 5b experiences the up-down process. In addition, with *N* are outside critical and $N \rightarrow \infty$, $\tau _{QSL}$ monotonically decreases to zero. As a result, an analysis of the vanishing QSL time can be similarly given. We are therefore capable of attributing it to the evolving speed $v_{TQSL}$ scaling with the system size, which is as crucial as the energy variance in the ground state case. At the same time, in conjuction with Fig. 4 we then conclude a similar correspondence relation between the QSL and the OC as zero-temperature ground state.

## Conclusions

We have applied the concept of the OC to the system composed of the nearest-interaction spins in order to investigate the relationship between the OC and QSL. The dynamical occurrence of the fidelity as well as the maximal rate of quantum evolution have been obtained by using the exactly solvable XY spin chain interacting with a single qubit impurity. We exhibit how the exponentially sensitive OCs are affected by the large *N*, and interestingly a similar exponential decay for QSL is also shown. Here we emphasize that the QSL specifies a universal bound of the fidelity between the initial state and the time evolved state. The numerical result reveals a striking similarity of the OC effect based on the fact that the perturbation forces the spin system to be an orthogonal state in the large *N* limit. In this respect, we quantitatively link the OC to the mechanism of quantum speedup characterized by the QSL time. For an initial ground state, we demonstrate that the OC effect manifests itself following by the energy variance scaling extensively with *N* and the vanishing QSL time due to a substantial relation between the emerging OC and the QSL time. We also investigate the finite temperature effect, where the fidelity and rate of quantum evolution are both suppressed by temperature, meaning that the minimum time to reach targeted state characterized by an up-down behaviour. Then the thresolds of temperature and *N* are obviously seen in the QSL time at finite temperatures. We have also proposed that the temperature-dependent $v_{TQSL}$ is as vital as the energy variance in initial ground case to the decisive relation between the OC phenomenon and the QSL time.
